# Optimizing health outcomes for older adults with sarcopenic obesity: a scoping review of nutrition, exercise and combined interventions

**DOI:** 10.3389/fnut.2026.1936660

**Published:** 2026-10-07

**Authors:** Zihan Li, Jiayu Wang, Xinyan Han, Yu Tian, Xinyu Zhu, Yijia Li, Lei Liu, Yikang Xu

**Affiliations:** 1 School of Nursing, Liaoning University of Traditional Chinese Medicine, Shenyang, Liaoning, China; 2 Nursing Department, Cancer Hospital of Dalian University of Technology, Shenyang, Liaoning, China; 3 Technical Faculty, Friedrich-Alexander-Universität Erlangen-Nürnberg, Nuremberg, Bayern, Germany; 4 Department of Cardiology, The Second Affiliated Hospital of Shenyang Medical College, Shenyang, Liaoning, China

**Keywords:** exercise intervention, nutritional intervention, obesity, older adults, review, sarcopenia, sarcopenic obesity (SO)

## Abstract

**Background:**

Against the backdrop of an aging population, sarcopenic obesity—a high-risk geriatric syndrome—significantly increases the risk of disability and mortality among older adults. However, current nutrition, exercise, and combined intervention programs for this population are diverse, with scattered outcome measures and a lack of systematic integration, severely limiting the identification and translation of optimal clinical practice strategies.

**Methods:**

This study was conducted from February to March 2026. Under the leadership of one researcher and with the assistance of two professional librarians, a search strategy was developed to conduct a systematic search of four Chinese databases: CNKI, Wanfang, VIP, and SinoMed, as well as four English-language databases: PubMed, Web of Science, CINAHL, and Embase. The search period spanned from the inception of each database to February 24, 2026.

**Results:**

This review included a total of 30 studies involving 8,587 obese older adults with sarcopenia. Interventions can be categorized into three major types: exercise training, nutritional intervention, and combined exercise-nutrition interventions. The included studies reported findings across five major domains: body composition, muscle strength and physical function, metabolic and cardiovascular risk, overall health and quality of life, and muscle mass and biomarkers. The existing literature is characterized by diverse intervention protocols and a wide range of outcome measures; some studies reported concurrent changes in body composition and muscle function indicators.

**Conclusion:**

This review systematically organized the classification of intervention measures and the distribution of outcome measures in studies on nutrition, exercise, and combined interventions for SO older adults, revealing the fragmented state of this field characterized by diverse intervention protocols and scattered outcome measures. Based on these findings, future research should focus on establishing a core set of outcome measures, conducting quantitative studies on the dose-response relationship of interventions, exploring the mechanisms of interaction between different exercise modalities and nutritional supplementation, and developing optimization strategies for personalized intervention plans, thereby providing an evidence-based foundation for the development of precise, localized intervention and care plans.

## 1 Introduction

Population aging has become a major public health issue of global concern. With declining fertility rates and increasing life expectancy, the population of older adults aged 60 years and above continues to rise worldwide. The World Health Organization (WHO) projects that by 2050, the global population of older adults will exceed 2.1 billion (1). As the country with the largest older adult population in the world, China faces a particularly severe aging situation. Data from the China Aging Affairs Development Report indicate that the number of older adults aged 60 years and above in China has reached 310 million, accounting for 22.0% of the total population, ranking first globally (2). The accelerated aging process is accompanied by a growing burden of non-communicable diseases, an increased risk of disability, and a diversification of health service needs (3), making it increasingly important to address healthy aging in China.

In this context, obesity, a key health concern among older adults highlighted by the World Health Organization (WHO) (4), has drawn increasing attention to its specific subtype, sarcopenic obesity. Sarcopenic obesity (SO) is defined as a pathological state characterized by the coexistence of sarcopenia and obesity, featuring a progressive decline in muscle mass and strength accompanied by abnormal accumulation of fat, particularly visceral fat (5). Globally, the prevalence of SO among older adults is approximately 11%, and this proportion reaches as high as 23% among those aged 75 years and above (6). The prevalence of SO among older adults in China is also concerning; research indicates that the incidence of SO among Chinese older adults is approximately 17.9%, with rates of about 21.4% in men and 12.5% in women, showing an increasing trend with age (7). SO is not merely a simple combination of sarcopenia and obesity; the two conditions interact through mechanisms such as insulin resistance, chronic low-grade inflammation, and oxidative stress, significantly exacerbating health impairments in older adults (8). Current evidence demonstrates that SO is closely associated with cardiovascular disease (9), type 2 diabetes mellitus (10), cognitive dysfunction (11), frailty (11), falls (12), and an increased risk of fractures (13), severely affecting not only the daily living abilities and quality of life of older adults but also leading to a significant increase in the risk of disability and all-cause mortality (14). Within the Chinese cultural context, the traditional belief that “it is hard to buy leanness in old age” remains deeply entrenched, leading older adults and their families to often regard weight loss or maintenance as a sign of health (15), while overlooking the potential harm of concurrent muscle loss and fat accumulation. This perception often results in SO being mistaken for a normal aging process, thereby delaying opportunities for early identification and intervention. Therefore, in-depth exploration and effective improvement of the current situation regarding SO among older adults within the Chinese cultural context has become a critical issue that must be addressed to achieve the strategic goals of healthy aging.

Currently, health authorities and researchers worldwide are actively exploring effective strategies to mitigate the adverse consequences of SO. Interventions targeting this population primarily encompass multiple dimensions such as exercise intervention, dietary intervention, surgical intervention, and pharmacological intervention (16). However, recent studies indicate that while weight management using novel pharmacological agents such as glucagon-like peptide-1 receptor agonists (GLP-1 RAs), including semaglutide, demonstrates significant efficacy in weight reduction among younger populations, simple weight loss in older adults may lead to further loss of muscle mass, thereby exacerbating the core issue of sarcopenia, thus limiting the effectiveness of pharmacological treatment in improving overall adverse outcomes in patients with SO (17). More importantly, within the Chinese cultural context, the traditional concept that “all medicines have their side effects” holds profound influence among older adult populations, resulting in widespread concerns and even resistance toward long-term medication use among older patients (18). The practical challenge of poor medication adherence, coupled with the potential risk of muscle loss associated with pharmacological interventions, has directed researchers' attention toward intervention pathways that are more acceptable to older adults and more congruent with their cultural background. Therefore, focusing on nutrition, exercise, and combined interventions for sarcopenic obesity in older adults within the Chinese cultural context carries considerable practical significance and research value.

Although the potential of nutrition, exercise, and combined interventions in the management of SO in older adults has been preliminary confirmed, current clinical research still exhibits a significant trend toward fragmentation. Nutrition, exercise, and combined interventions and their corresponding outcome indicators for older adults with SO remain dispersed and have not been systematically integrated. This fragmentation is primarily manifested in the diversity of intervention protocols, which hinders the extraction of optimal evidence, and the heterogeneity of evaluation criteria for outcome measures, which makes it difficult to establish a standardized evaluation system. Therefore, this study employs a scoping review methodology, aiming to systematically summarize and synthesize the implementation protocols of nutrition, exercise, and combined interventions in the management of SO in older adults has been preliminary confirmed, current clinical research still and various health outcome indicators for older patients with SO, in order to provide scientific empirical support for the future development of precise and context-specific non-pharmacological care strategies for older adults with SO, thereby contributing to the health and wellbeing of older adults in their later years and advancing the goal of healthy aging.

## 2 Materials and methods

This review aims to summarize and synthesize nutrition, exercise, and combined interventions in the management of SO in older adults has been preliminary confirmed, current clinical research still and their associated health outcome indicators for older patients with SO, and further identify existing research gaps in this field. As a method suitable for evidence synthesis and integration, integration, and summary, a scoping review can be effectively applied to topics that have not yet been extensively investigated, helping researchers understand the breadth and depth of existing research, thereby revealing gaps in the literature (19). This review strictly adheres to the Preferred Reporting Items for Systematic Reviews and Meta-Analyses Extension for Scoping Reviews (PRISMA-ScR) framework recommended by the Joanna Briggs Institute (JBI) Evidence-Based Healthcare Center (20). This framework is derived from the five-stage methodological framework for scoping reviews proposed by Arksey and O'Malley (21) and has been subsequently developed and evolved from it.

### 2.1 Research questions

This review employs a scoping review methodology to investigate nutrition, exercise, and combined intervention, implementation methods, and associated health outcomes in older patients with SO. The study addresses the following research questions: (1) What are the nutrition, exercise, and combined interventions for older patients with SO? (2) What health outcomes can be improved by nutrition, exercise, and combined interventions in older patients with SO?

### 2.2 Literature search strategy

To ensure the scientific rigor and comprehensiveness of the search strategy, two medical librarians were invited to participate in strategy development during the initial phase of this study. Based on their recommendations, a preliminary search was conducted in CNKI and PubMed to evaluate the effectiveness and appropriateness of the retrieval strategy through analysis of the obtained Chinese and English literature. During the formal search phase, the first author (Z.H.L.) systematically searched the following eight databases according to the finalized search strategy: English databases including PubMed, Web of Science Core Collection, CINAHL, and Embase; Chinese databases encompassing CNKI, Wanfang Database, VIP Database, and SinoMed. The detailed search strategies are presented in Appendix A. The search period covered from the inception of each database up to 24 February 2026, the search was restricted to studies published in Chinese and English. To ensure the breadth and completeness of literature coverage, no restrictions were imposed on outcome measures or study design types during the search process. Furthermore, to enhance the representativeness of the included literature, the researchers employed a snowball sampling method to screen and retrospectively search the reference lists of the included studies.

The literature search process was conducted under the guidance and supervision of the corresponding author (Y.K.X. and L.L.). For gray literature, the researchers identified and supplemented it through manual checking of catalogs. For literature management, the search results were first imported into EndNote 21 (Clarivate Analytics, PA, USA) for duplicate removal. Subsequently, two researchers (Z.H.L. and X.Y.H.) independently performed preliminary screening by reviewing titles and abstracts according to the predefined inclusion and exclusion criteria. For studies deemed “potentially relevant”, two additional researchers (J.Y.W. and X.Y.Z.) were invited to participate in discussions until a consensus was reached. After the preliminary screening was completed, the relevant literature was imported into Zotero 7.0.16 for further review through full-text reading, ultimately determining the final set of studies included in this review.

### 2.3 Inclusion and exclusion criteria

The inclusion criteria for this study were based on the Participant-Concept-Context (PCC) framework recommended by the JBI (22). (1) Participants: Older adults aged ≥60 years with a confirmed diagnosis of sarcopenic obesity (23). Given that there are currently no internationally unified diagnostic criteria for sarcopenic obesity, this review accepts the following three categories of diagnostic criteria based on a literature review: (a) Component-based diagnostic criteria: These criteria follow a two-step diagnostic logic of “sarcopenia first, obesity second”: Step 1: Sarcopenia is diagnosed based on low muscle mass according to the EWGSOP (24) or AWGS 2019 consensus (25), in conjunction with the presence of at least one of the following: low muscle strength or low physical function. Low muscle mass is determined using DXA (men < 7.0 kg/m^2^, women < 5.4 kg/m^2^) or BIA (men < 7.0 kg/m^2^, women < 5.7 kg/m^2^) (26); low muscle strength is defined as a grip strength of < 28.0 kg (men) or < 18.0 kg (women); impaired physical function is defined by a gait speed < 1 m/s, a time of ≥ 12 s for the 5-repetition chair stand test, or an SPPB score ≤ 9; Step 2: Based on the above, determine the presence of obesity according to the criteria proposed in the ESPEN-EASO 2022 Consensus (27) (BMI ≥ 30 kg/m^2^, body fat percentage ≥ 25% for men and ≥ 35% for women, waist circumference ≥ 102 cm for men and ≥ 88 cm for women, visceral fat area ≥ 100 cm^2^). Individuals who meet both of the above criteria are diagnosed with sarcopenic obesity. Studies using these criteria must clearly report the specific cutoff values employed. (b) Composite index diagnostic criteria—these integrate muscle and fat parameters into a continuous score to assess the severity of sarcopenic obesity (SO), such as the Sarcopenic Obesity Index (SOI) (28) and the Sarcopenia Z-Score (29). The advantage of this type of criterion is that it does not rely on binary cutoff values and is more sensitive to the identification of early-stage or mild SO (i.e., during the transitional stage of SO development). Studies using these criteria must clearly report the calculation formula for the index, the source of the parameters, and the diagnostic thresholds. (c) Combined body composition diagnostic criteria—based on body composition data measured by DXA or BIA, SO is defined as the coexistence of “low skeletal muscle mass index (SMI)” and “high body fat percentage” or “high fat mass index.” For example: SMI below the cutoff values recommended by the AWGS 2019 (25) (DXA: men < 7.0 kg/m^2^, women < 5.4 kg/m^2^; BIA: men < 7.0 kg/m^2^, women < 5.7 kg/m^2^), combined with a body fat percentage of ≥25% for men and ≥35% for women, or a fat mass index of ≥9.0 kg/m^2^ for men and ≥12.0 kg/m^2^ for women (27). Studies using these criteria must clearly report the measurement methods and cutoff values for muscle and fat mass. All studies included in this review must explicitly describe their subjects as having “sarcopenic obesity” or meeting the diagnostic criteria for both sarcopenia and obesity. Studies that report only “osteoporotic obesity” without addressing the diagnostic criteria for sarcopenia were excluded; however, studies in which participants had concurrent diagnoses of sarcopenia and osteoporosis and met any of the above SO diagnostic criteria (such as sarcopenia-osteoporosis or obesity syndrome with reduced bone and muscle mass) were included. (2) Concept: The interventions addressed in this review are defined as non-pharmacological management strategies centered on exercise training, nutritional guidance, or a combination of both. Specifically, these include: (1) exercise training (e.g., resistance training, aerobic training, circuit training, whole-body electrical muscle stimulation, etc.); (2) nutritional interventions (e.g., dietary behavior modification, protein supplementation, multivitamin, and mineral supplements, etc.); (3) combined exercise-nutrition interventions (e.g., resistance training combined with protein supplementation, whole-body electrical muscle stimulation combined with protein supplementation, etc.). It should be noted that this approach aims to comprehensively present the diversity of SO intervention studies and provide exploratory evidence for future research. (3) Context: Participants living in any setting, including the community, hospitals, or at home. (4) Study Types: Included literature must consist of original research that has been peer-reviewed and formally published in Chinese or English academic journals. Study designs include, but are not limited to, randomized controlled trials, cross-sectional studies, cohort studies, and case-control studies. Exclusion Criteria: (1) Duplicate publications. (2) Literature for which the full text is unavailable. (3) Case reports, conference proceedings, reviews, theses, books, newspapers, and experimental protocols.

### 2.4 Data extraction

During the integration phase of the included literature, two researchers (Z.H.L. and J.Y.W.) collaboratively developed a standardized data extraction form, which was agreed upon by all authors, to systematically extract literature information and standardize key terms involving Eastern and Western cultural differences, ensuring data consistency and comparability. The design and refinement of all charts were completed by one author (X.Y.H.) and approved by all authors. The specific information extracted included literature authors, publication year, country of origin, study design type, target population characteristics, sample size, intervention modality, intervention duration, and effective outcome measures. Data organization was entered and managed using Microsoft Excel software. The entire process was conducted under the guidance of the corresponding author (Y.K.X. and L.L.). In the event of disagreements during data extraction or categorization, two additional researchers (Y.T. and Y.J.L.) were invited to participate in comparison and discussion until a consensus was reached. To ensure the accuracy and cultural appropriateness of terminology, the final data results were reviewed by a researcher on the team (Y.J.L.) who has overseas experience and is proficient in English terminology, thereby maximizing the avoidance of semantic bias or ambiguous expressions arising from Eastern and Western cultural differences. When full texts were unavailable or key information was missing, corresponding authors were contacted *via* email to request additional data; if no response was received after two follow-up emails, the study was excluded or the missing information was noted as unavailable. According to the JBI scoping review methodology guidelines and the PRISMA-ScR reporting framework, scoping reviews aim to map existing evidence rather than synthesize effect sizes of interventions; therefore, this study did not conduct a formal assessment of bias risk or quality rating for the included primary studies.

## 3 Results

### 3.1 Search results and screening process

The initial search yielded a total of 2,976 records, and no additional eligible studies were identified through the snowballing method. After deduplication, 266 duplicate records were removed, leaving 2,710 records for the initial screening phase. Following screening by title and abstract, 2,621 irrelevant records were excluded, resulting in 89 records proceeding to the full-text screening stage. After further assessment through full-text reading, 57 records were excluded for not meeting the inclusion criteria, ultimately resulting in 30 studies being included in this review. The detailed literature screening process is presented in Figure 1 (Appendix B1). It is worth noting that during the pre-search phase, a pilot search was conducted in the Cochrane Library, yielding 344 records; however, the full texts of these records could not be obtained from this database, and all such records were already covered by the other databases included in this study. Therefore, the Cochrane Library was not included as a search source in the formal search process.

**Figure 1:**
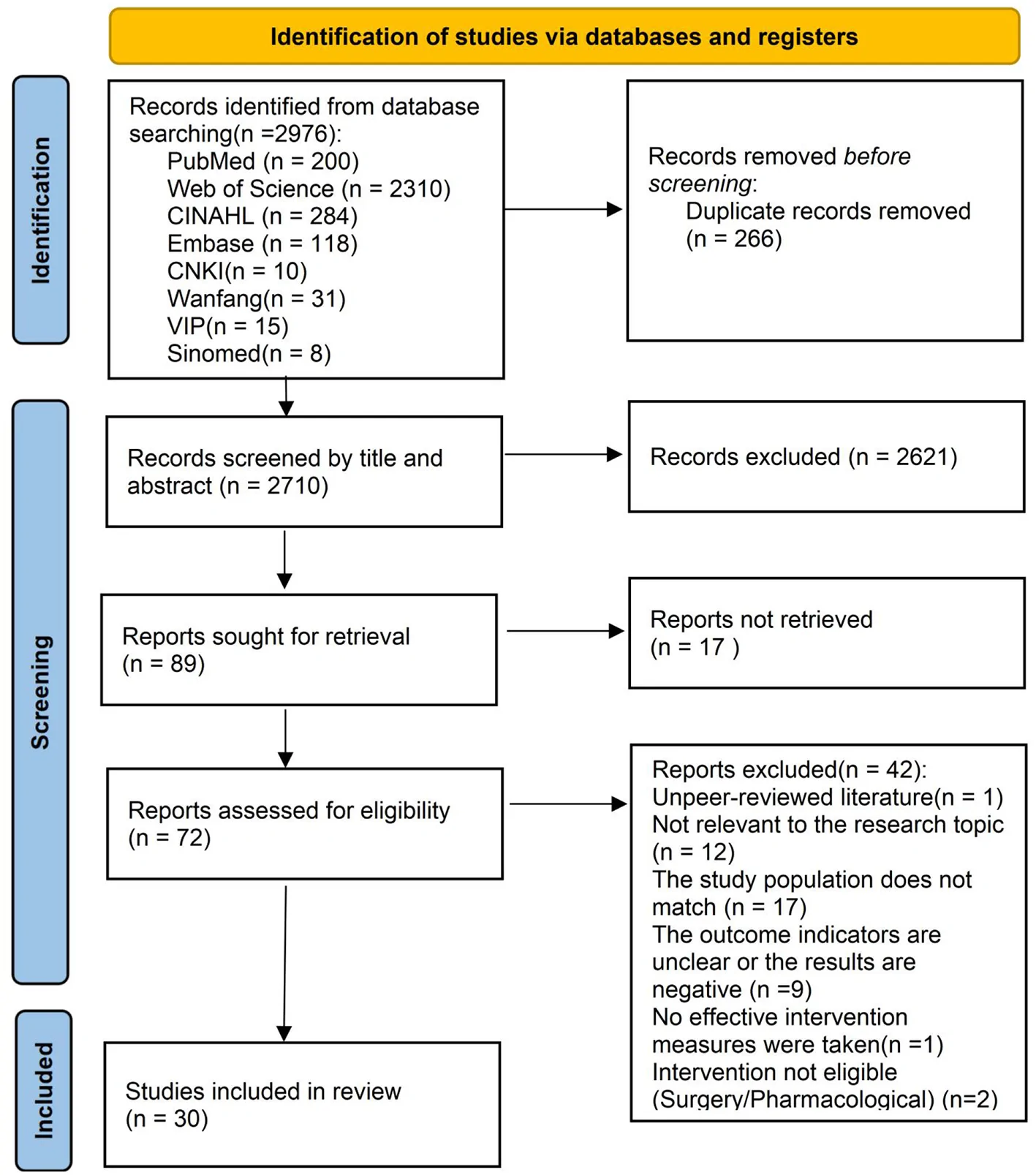
PRISMA flow-diagram of included studies.

### 3.2 Basic characteristics of included studies

Based on the 30 included studies, a total of 8,587 older adults with SO were involved. Examining the link between study design and population characteristics revealed a clear hierarchical pattern in the existing evidence. The 28 interventional studies, comprising 27 randomized controlled trials (RCTs) and one quasi-experimental study, primarily targeted older adults with confirmed SO, aiming to validate the effectiveness of specific exercise or nutrition regimens. In contrast, the two observational studies (cohort study) focused on the general older population, exploring the developmental trajectory between physical activity levels, eating behaviors, and SO risk. This distribution suggests that SO research is currently transitioning from “risk factor exploration” to “clinical intervention validation,” yet stratified intervention evidence for specific subgroups, for example, those with varying SO severity, remains insufficient.

Geographically, the studies covered 13 countries and regions worldwide. China contributed the most studies (8, including Mainland China and Taiwan), followed by Germany (6), Brazil (6), South Korea (3), the United States (2), and 1 each from Japan, Iran, Mexico, Spain, Canada, and Tunisia. This pattern indicates that SO has become a globally recognized public health concern, though research priorities may differ across regions. Regarding study types, RCTs dominated (27 studies), with additional cohort studies (2) and one quasi-experimental study. To present the evidence map clearly, this review stratified findings by design type: interventional evidence, the 27 RCTs and one quasi-experimental study provided core evidence on exercise, nutrition, combined interventions, and their health outcomes; observational evidence, the two cohort studies offered epidemiological data on physical activity levels, eating speed, and disease risk in older adults with SO. These observational and non-randomized controlled designs enriched the research perspective, helping elucidate the natural course and long-term impact of SO from multiple dimensions. Figure 2 displays the study types and geographic distribution. Regarding publication timeline, the included studies spanned 2014 to 2025, showing an overall upward trend. The period from 2016 to 2018 marked initial development, with a notable increase after 2019. The year 2025 recorded the highest publication volume, with seven included studies. Table 1 presents detailed characteristics.

**Figure 2:**
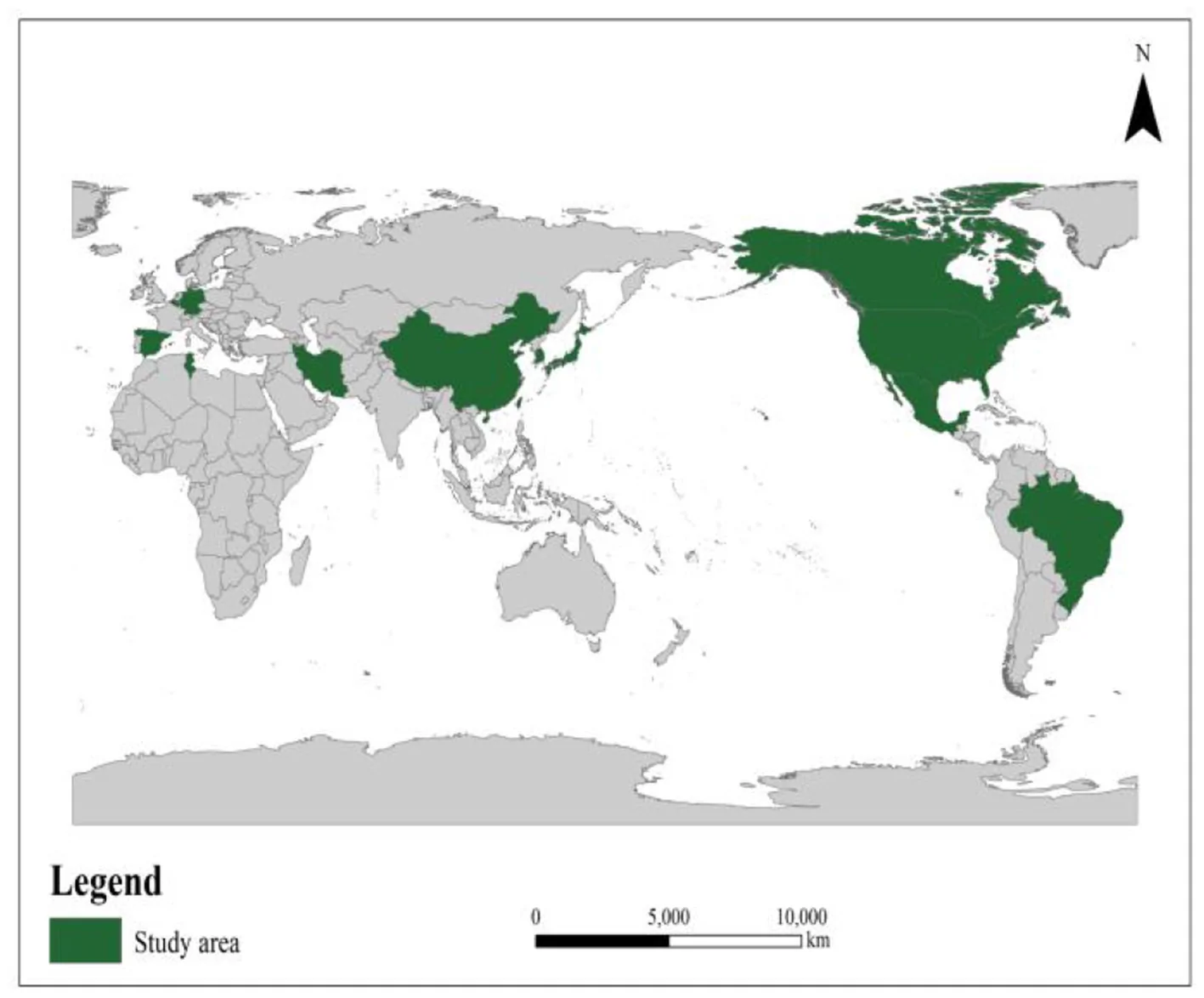
The global distribution and types of studies included in the literature.

**Table 1:** Table of feature extraction for included literature.

References	Country	Design	Objective	Sample size	Means of intervention	Duration of intervention	Reported outcome measures
Balachandran et al. (41)	U.S.A.	RCT	Sarcopenic obesity older adults (aged ≥ 60)	*N* = 21	High-speed circuit training (HSC)	15 weeks	Lower body power, Rating of perceived exertion (RPE), physical function, body fat percentage, sitting and standing speed
Gadelha et al. (34)	Brazil	RCT	Sarcopenic obesity older women (aged 60–80)	*N* = 133	Progressive resistance training (RT)	24 weeks	Fat-free mass (FFM), sarcopenic obesity index, body fat percentage
Kemmler et al. (52)	Germany	RCT	Community-dwelling sarcopenic obesity older women (aged ≥ 70)	*N* = 75	1.Whole-body electromyostimulation (WB-EMS) 2.WB-EMS combined with protein supplementation	26 weeks	Sarcopenia Z-score, skeletal muscle mass index (SMI), gait speed, body fat percentage, grip strength
Kim et al. (58)	Japan	RCT	Community-dwelling sarcopenic obesity older women (aged ≥ 70)	*N* = 139	Exercise combined nutrition	3 months	Body fat mass, trunk fat mass, appendicular fat mass, knee extension strength, gait speed, average number of steps, leptin, body fat percentage
Vasconcelos et al. (35)	Brazil	RCT	Sarcopenic obesity older women (aged 65–80)	*N* = 31	Progressive resistance training (RT)	10 weeks	Knee extensor muscle power
Wittmann et al. (53)	Germany	RCT	Community-dwelling sarcopenic obesity older women (aged ≥ 70)	*N* = 75	WB-EMS combined with protein supplementation	26 weeks	Mean arterial pressure, waist circumference, High-density lipoprotein cholesterol (HDL-C), metabolic syndrome Z-score,
Chen et al. (37)	Taiwan, China	RCT	Sarcopenic obesity older adults (aged 65–75)	*N* = 93	Resistance training (RT)	8 weeks	Body fat percentage, Body fat mass (BFM), Visceral fat area (VFA), Waist circumference
Huang et al. (36)	Taiwan, China	RCT	Sarcopenic obesity older women (aged ≥ 60)	*N* = 35	Progressive resistance training (RT)	12 weeks	Right upper extremity fat mass, total fat mass, body fat percentage
Kemmler et al. (54)	Germany	RCT	Community-dwelling sarcopenic obesity older women (aged ≥ 70)	*N* = 100	WB-EMS combined with protein supplementation	16 weeks	Sarcopenia Z-score, body fat percentage, Skeletal muscle mass index (SMI), handgrip strength, waist circumference
Liao et al. (30)	Taiwan, China	RCT	Sarcopenic obesity older women (aged 60–80)	*N* = 56	Elastic band resistance training (ERT)	12 weeks	Body fat percentage, total skeletal muscle mass (TSM), Lean mass index (LMI), Appendicular lean mass index (AMI)
de Oliveira Silva et al. (38)	Brazil	RCT	Sarcopenic obesity older women (aged ≥ 60)	*N* = 66	Interval resistance training	16 weeks	Body fat percentage, body fat mass, Waist circumference (WC), Waist-to-hip ratio (WHr), Neck circumference (NC), visceral fat area, trunk fat mass
Kemmler et al. (55)	Germany	RCT	Community-dwelling sarcopenic obesity older men (aged ≥ 70)	*N* = 100	WB-EMS combined with protein supplementation	16 weeks	Appendicular skeletal muscle mass, trunk fat mass, leg-extensor strength, gait velocity
Kemmler et al. (56)	Germany	RCT	Community-dwelling sarcopenic obesity older men (aged ≥ 70)	*N* = 100	WB-EMS combined with protein supplementation	16 weeks	Body fat mass, Trunk fat mass (TF), Waist circumference (WC), Visceral fat area (VFA)
Liao et al. (31)	Taiwan, China	RCT	Sarcopenic obesity older women (aged 60–80)	*N* = 63	Elastic band resistance training (ERT)	12 weeks	Total skeletal mass (TSM), Appendicular lean mass (ALM), Appendicular muscle index (AMI), Lean mass index (LMI), Skeletal muscle mass index (SMI), Muscle quality (MQ), Gait speed (GS)
Nabuco et al. (59)	Brazil	RCT	Sarcopenic obesity older women (aged ≥ 60)	*N* = 26	Resistance training combined with protein supplementation	12 weeks	Appendicular lean soft tissue (ALST), body fat mass, trunk fat mass, interleukin-6 (IL-6), body fat percentage
Banitalebi et al. (32)	Iran	RCT	Sarcopenic obesity older women (aged 65–80)	*N* = 63	Elastic band resistance training (ERT)	12 weeks	Muscle quality (MQ), sarcopenia Z-score, handgrip strength, estradiol 2, leptin, activities of daily living (ADL), instrumental activities of daily living (IADL)
Kemmler et al. (57)	Germany	RCT	Community-dwelling sarcopenic obesity older men (aged ≥ 70)	*N* = 100	WB-EMS combined with protein supplementation	16 weeks	Body fat mass, waist circumference, trunk fat mass, triglyceride, skeletal muscle mass index (SMI)
Ribeiro Santos et al. (42)	Brazil	Cohort study	Sarcopenic obesity older adults (aged 60–97)	*N* = 395	Sufficient physical activity (Baecke physical activity questionnaire)	24 months	Skeletal muscle mass index (SMI), handgrip strength, body fat percentage
Gutiérrez-López et al. (43)	Mexico	RCT	Sarcopenic obesity older women (aged ≥ 60)	*N* = 60	Moderate intensity exercise program	3 months	Muscle strength, gait speed
Jung et al. (40)	South Korea	RCT	Sarcopenic obesity older women (aged ≥ 65)	*N* = 30	Circuit Exercise Training	12 weeks	Body mass index (BMI), body fat percentage, Fat free mass (FFM), Waist-to-hip ratio (WHr), heart rate, systolic blood pressure, high-density lipoprotein cholesterol (HDL-C), high sensitivity C-reactive protein, interleukin-6
Meng et al. (44)	China	RCT	Sarcopenic obesity older adults (aged ≥ 60)	*N* = 60	Aerobic combined with resistance exercise	12 weeks	Grip strength, body fat percentage, visceral fat area, serum albumin, quality of life, appendicular skeletal muscle mass
Ferhi et al. (45)	Tunisia	RCT	Sarcopenic obesity older adults (aged ≥ 65)	*N* = 72	Posture, Strengthening, and motricity (PSM) program	24 weeks	Lean body mass (LBM), Fat body mass (FBM), Handgrip force, Gait speed
Yin et al. (49)	China	RCT	Community-dwelling sarcopenic obesity older adults (aged ≥ 60)	*N* = 60	Dietary behavior Intervention	15 weeks	Body weight, dietary quality
Jung et al. (39)	South Korea	RCT	Sarcopenic obesity older women (aged ≥ 65)	*N* = 30	Circuit exercise training (HSC)	12 weeks	Body fat mass, body fat percentage, free-fat mass
Nilsson et al. (61)	Canada	RCT	Sarcopenic obesity older men (aged ≥ 65)	*N* = 179	Multi-ingredient supplementation (MIS)	12 weeks	Appendicular lean mass (ALM), body fat percentage, Appendicular lean Mass index (AMI), grip strength
Álvarez-Bustos et al. (46)	Brazil	Quasi-experimental study	Community-dwelling sarcopenic obesity older adults (aged ≥ 60)	*N* = 78	Multicomponent exercise training(MCET)	1 year	Balance, Gait speed (GS)
Guo et al. (33)	China	RCT	Sarcopenic obesity older women (aged 60–70)	*N* = 30	Elastic band resistance training (ERT)	36 weeks	Body fat percentage, grip strength, sitting and standing speed
Han et al. (60)	China	RCT	Sarcopenic obesity older adults type 2 diabetic patients with sarcopenic obesity (aged 60–75)	*N* = 75	Resistance training combined with whey protein supplementation (RT+WPS)	12 weeks	HbA1c, handgrip strength, sitting and standing speed, appendicular skeletal muscle mass
Lee et al. (50)	South Korea	Cohort study	Sarcopenic obesity older adults (aged ≥ 65)	*N* = 6,202	Slow eating speed	16-year follow-up	Waist circumference, BMI
Polo-Ferrero et al. (47)	Spain	RCT	Community-dwelling Sarcopenic obesity older women (aged ≥ 65)	*N* = 40	Power training (PT)	32 weeks	Skeletal muscle mass index (SMI), Appendicular skeletal muscle mass (ASM), Body fat percentage, Visceral fat area (VFA), Waist circumference (WC), Gait speed (GS), BMI

### 3.3 Nutrition, exercise, and combined intervention approaches for older adults with SO

This study synthesized 30 studies and identified three intervention categories for older adults with SO: exercise training interventions, nutrition interventions, and combined exercise nutrition interventions.

#### 3.3.1 Exercise training interventions

Among the 30 included studies, resistance training was the most frequently mentioned intervention modality. Specifically, four studies employed elastic band resistance training to improve health outcomes in older patients with SO (30–33); three studies adopted progressive resistance training (34–36); and conventional resistance training (37) and periodised resistance training (38) were each adopted by one study. Circuit training was also employed in three studies, of which two employed circuit training as an intervention modality (39, 40), and one study utilized high-speed circuit (HSC) training (41). In addition, adequate physical activity (42), moderate-intensity exercise (43), combined aerobic and resistance exercise (44), posture, strength and movement training (45), multicomponent exercise training (46), and strength/power training (47) were each investigated in one study and demonstrated positive effects.

#### 3.3.2 Nutritional interventions

Nutritional and dietary interventions represent one of the important intervention strategies for SO (48). Among the 30 included studies, two studies mentioned nutrition-related interventions, of which dietary behavior intervention (49) and slow eating speed (50) were each investigated in a single study.

#### 3.3.3 Combined exercise and nutritional interventions

The combination of exercise and nutritional interventions is an effective approach for improving muscle mass and alleviating symptoms of sarcopenia (51). Among the 30 included studies, six studies employed whole-body neuromuscular electrical stimulation combined with protein supplementation to improve health outcomes in older adults with SO (52–57), particularly for addressing sarcopenia symptoms. Additionally, one study adopted exercise combined with nutritional intervention (58), two studies employed resistance training combined with protein supplementation (59, 60) as an effective intervention modality, and one study involved resistance training combined with multicomponent nutritional supplements (61).

### 3.4 Outcome measures reported in studies on nutrition, exercise, and combined interventions for older adults with SO

Based on the 30 included studies, this review identified five categories of outcome measures reported in non-pharmacological intervention studies for older adults with SO. This section describes the types and distribution of outcome measures measured and reported in the included literature.

#### 3.4.1 Body composition indicators

Body composition is central to the diagnosis of SO and constitutes the most direct indicator when evaluating intervention efficacy. Among the included studies, a total of 24 studies reported changes in body composition, encompassing muscle-related indicators and fat-related indicators. Regarding fat-related indicators, 16 studies confirmed the effectiveness of nutrition, exercise, and combined interventions on body fat percentage (30, 33, 34, 36–41, 44, 47, 52, 58, 59, 61); four studies addressed fat-free mass/lean body mass (34, 39, 40, 45); eight studies involved body fat mass (BFM) (37–39, 45, 56–59); five studies mentioned visceral fat area (VFA) (37, 38, 44, 47, 56); waist circumference (WC) was also used as an outcome indicator in eight studies (37, 38, 47, 50, 53, 54, 56, 57); six studies validated the effectiveness on trunk fat mass (38, 55–59); two studies involved waist-to-hip ratio (WHr) (38, 40); and neck circumference (NC) (38) and limb fat mass (58) were each mentioned in one study. Regarding muscle-related indicators, seven studies confirmed the effectiveness on skeletal muscle mass index (SMI) (30, 31, 42, 47, 52, 54, 57); seven studies involved appendicular skeletal muscle mass/appendicular lean mass (ALM) (31, 44, 47, 55, 58, 60, 61); three studies involved appendicular muscle mass index (AMI) (30, 31, 61); two studies mentioned lean mass index (LMI) (30, 31); and three studies mentioned the improvement of sarcopenia Z-score through nutrition, exercise, and combined interventions (32, 52, 54). One study also focused on the inverse change in right upper limb fat mass (36). Additionally, one studies recorded the effectiveness of nutrition, exercise, and combined interventions on body weight (49); and three studies identified the improvement effect of nutrition, exercise, and combined interventions on body mass index (BMI) in older patients with SO (40, 47, 50).

#### 3.4.2 Muscle strength and physical function indicators

Muscle strength and physical function are key factors in predicting the risk of disability and prognosis in older adults. In this review, 29 studies confirmed that nutrition, exercise, and combined interventions significantly improved 10 types of muscle strength and physical function indicators in older patients with SO. Regarding muscle strength indicators, handgrip strength was the most frequently cited strength indicator, showing significant improvement in nine studies (32, 33, 42, 44, 47, 52, 54, 60, 61). Additionally, lower limb power/knee extensor power and lower limb extensor strength were incorporated as important indicators for assessing lower limb explosive power in three studies (35, 41, 55) and one study (55), respectively. Regarding physical function, nutrition, exercise, and combined interventions significantly improved patients' mobility and balance. Eight studies reported the effectiveness of interventions on gait speed (GS) (31, 43, 44, 46, 47, 52, 55, 58). Five-times sit-to-stand test time and balance ability were also common functional assessment indicators, validated in three studies (33, 41, 60) and one study (46), respectively. Regarding activities of daily living, some studies further focused on the impact of interventions on patients' actual functional capacity, with one study addressing activities of daily living and IADL (32), and another study involving average daily step count (58). Furthermore, one study used the rating of perceived exertion (RPE) to assess changes in exercise tolerance (41).

#### 3.4.3 Metabolic and cardiovascular risk indicators

SO is often accompanied by metabolic disturbances and increased cardiovascular risk. Nine studies explored the regulatory effects of non-pharmacological interventions on eight types of related metabolic indicators. Regarding lipid and glucose-related indicators, two studies involved high-density lipoprotein cholesterol (HDL-C) (40, 53); one study involved triglycerides (57) and glycated hemoglobin (60) were each included as outcome indicators in one study. Regarding blood pressure and metabolic syndrome-related indicators, one study validated the effectiveness of non-pharmacological interventions on systolic blood pressure and heart rate (40), and another study explored the impact of nutrition, exercise, and combined interventions on mean arterial pressure and metabolic syndrome Z-score (53).

#### 3.4.4 Comprehensive health and quality of life indicators

Beyond physiological indicators, patients' subjective health perception and overall quality of life are also important dimensions for evaluating intervention success. Three studies reported two types of such outcomes. Regarding quality of life indicators, quality of life showed significant improvement in one studies (44). Regarding dietary and behavioral indicators, one study reflected that improvements in dietary quality demonstrated the direct effects of nutritional intervention (49).

#### 3.4.5 Muscle mass and biomarkers

To further elucidate the mechanisms of intervention effects, 12 studies examined seven types of biomarkers reflecting intrinsic muscle quality and molecular-level changes. Muscle quality (MQ), as an indicator for assessing muscle functional efficiency, was improved in two studies (31, 32). Non-pharmacological interventions significantly reduced chronic low-grade inflammation levels, with two studies reporting decreases in high-sensitivity C-reactive protein (hs-CRP) (39, 40) and interleukin-6 (IL-6) (40, 59), respectively. Two studies mentioned leptin (32, 58); one study each mentioned oestradiol (32) and insulin-like growth factor-1 (IGF-1) levels (40); additionally, one study validated the effectiveness of nutrition, exercise, and combined interventions on serum albumin (44).

## 4 Discussion

This review synthesized 30 studies involving 8,587 obese older adults with SO, identifying three types of interventions (exercise training, nutritional intervention, and combined exercise-nutritional intervention) and five outcome domains. Resistance training interventions (particularly resistance training with elastic bands) showed the highest consistency in reported outcomes for body composition and muscle function (13 out of 15 studies reported a decrease in body fat percentage, and 9 out of 11 studies reported an increase in grip strength). This was primarily due to the larger number of studies (15) and the advantage of a predominantly randomized controlled trial (RCT) design (14 out of 15 were RCTs).Combined exercise-nutrition interventions [particularly whole-body electrical muscle stimulation (WB-EMS) combined with protein supplementation] reported the broadest coverage of outcomes in terms of simultaneously improving body composition, muscle function, and metabolic indicators; however, it should be noted that six of these studies were conducted by the same German research team, limiting the independence of the evidence (52–57). However, significant heterogeneity in intervention protocols and outcome measures (over 30 outcome measures reported across 30 studies), as well as low reporting rates for dosage parameters (only 40% reported protein dosage and 30% reported training intensity), highlight critical gaps in the current evidence base.

### 4.1 Implementation methods and effects of exercise training interventions

The included studies consistently reported an association between resistance training with elastic bands and improved health outcomes in older adults with sarcopenic obesity. In terms of intervention protocols, most studies employed a frequency of two to three sessions per week, with multi-set training targeting major muscle groups in the upper and lower extremities; among these, the 36-week personalized program implemented by Guo et al. (33) stood out for its 36-week personalized program. This study strictly adhered to the FITT-VP principles (frequency, intensity, time, type, volume, and progression) and used heart rate monitoring to maintain intensity between 60 and 80% of maximum heart rate, thereby achieving precise load management over the long term. In contrast, the 12-week interventions conducted by Liao et al. (30, 31) and Banitalebi et al. (32) reported immediate benefits of short-term intensive training; the former further reported the persistence of these effects during a 9-month follow-up period. Regarding improvements in outcome measures, the four studies demonstrated highly consistent positive effects (30–33). In terms of body composition, both long-term and short-term resistance band training interventions significantly reduced body fat percentage and increased the skeletal muscle mass index of the limbs, effectively reversing the “low muscle, high fat” sarcopenic obesity phenotype. In terms of functional ability, patients' grip strength, gait speed, and lower-limb strength all improved significantly. Some studies showed an average increase of more than five repetitions in the number of chair stands, indicating a reduced risk of falls among older adults 57. More importantly, the long-term (36-week) resistance band training program by Guo et al. (33) demonstrated significant advantages in improving muscle function. The study found that long-term interventions significantly improved patients' grip strength and lower-limb function (sit-to-stand speed), suggesting that a sufficiently long intervention period may be key to achieving functional benefits for obese patients with sarcopenia. Compared to short-term interventions, long-term training may more effectively promote muscle functional adaptation through more sustained mechanical load stimulation; however, this requires further confirmation in future studies through the measurement of relevant biological markers.

Progressive resistance training, through the gradual increase in load intensity and training volume, provides a systematic exercise intervention program for older adults with sarcopenic obesity. Gadelha et al. (34) conducted a 24-week progressive resistance training program involving 133 elderly women with SO, training three times weekly with loads gradually increasing from 60 to 80% of 1-RM. The training program included multi-joint exercises such as bench press, leg press, and knee flexion and extension. The results showed that the intervention group experienced a significant increase in total FFM and AFFM, a 21% improvement in the SO index, and a significant increase in peak isokinetic torque at the knee joint, whereas the control group exhibited a downward trend in all of these indicators. Vasconcelos et al. (35) conducted a 10-week progressive resistance training program for 28 elderly female patients with SO. The first 4 weeks focused on endurance training at low loads, and high-speed components were introduced starting in the 5 week. However, no significant improvements were observed in knee extensor power, knee extensor strength, fatigue index, SPPB, or gait speed after the intervention, suggesting that short-term training may struggle to induce the expected functional benefits in this population. Huang et al. conducted a 12-week progressive resistance training program using elastic bands in 35 elderly female patients with SO. The study observed significant decreases in right upper limb fat mass, total fat mass, and body fat percentage, as well as significant increases in BMD, T-score, and Z-score. These studies indicate that progressive resistance training may be associated with improvements in body composition, muscle strength, and physical function, but requires a longer intervention period. Gadelha et al. (34) employed a 24-week long-term program that yielded significant improvements in FFM, AFFM, the SO index, and PT, whereas Vasconcelos et al. (35) observed no functional benefits with their 10-week program, suggesting that older adults with SO may require a longer training period to achieve cumulative effects on muscle protein synthesis. It is worth noting that in the study by Gadelha et al. (34), FFM increased significantly while fat mass (FM) did not decrease significantly; in the study by Huang et al. (36), upper-body fat mass decreased but muscle mass did not increase significantly. This suggests that the effects of progressive resistance training may be site-specific: upper-body fat is more sensitive to resistance stimulation, whereas lower-body muscle growth requires a longer adaptation period.

The effectiveness of phased resistance training as an intervention for elderly patients with sarcopenic obesity has certain limitations. de Oliveira Silva et al. (38) conducted a 16-week phased resistance training program for eight elderly women with sarcopenic obesity, twice weekly, with a periodized training load design: the first 4 weeks consisted of 12–14 maximum repetitions, followed by a gradual reduction every 4 weeks to 10–12, 8–10, and 6–8 maximum repetitions, respectively. The results showed that the intervention group exhibited significant improvements from baseline only in 45° leg press strength and elbow flexion repetitions, with improvement rates of 40.5 and 21.4%, respectively; however, no significant improvements were observed in body composition indicators such as body fat percentage, waist circumference, waist-to-hip ratio, and neck circumference, nor were there any notable changes in physical function indicators such as the 30-s sit-to-stand test and the timed up-and-go test (38). It is worth noting that the non-SO control group, which underwent the same training during the same period, achieved significant improvements in all of the aforementioned indicators, with effect sizes generally higher than those of the SO group (38). This study suggests that patients with sarcopenic obesity may respond less effectively to phased resistance training than non-sarcopenic individuals, with improvements primarily concentrated in local muscle strength rather than body composition and overall function (38). This phenomenon may be related to chronic inflammation, intramuscular lipid infiltration, and anabolic resistance associated with obesity (62), suggesting that patients with sarcopenic obesity may require higher training frequencies or longer intervention periods to achieve comprehensive benefits.

Circuit training and high-speed circuit training (HSC) provide effective intervention strategies for elderly patients with SO by alternating resistance training and aerobic exercise and shortening rest periods. Jung et al. (40) conducted a 12-week HSC program for 28 elderly female patients with SO, three times per week, with training consisting of alternating six resistance exercises and two aerobic exercises, with loads controlled between 60 and 80% of 1-RM.Results showed that the intervention group exhibited significant improvements in body fat mass, body fat percentage, heart rate, pulse pressure product, ankle-brachial pulse wave velocity, and interleukin-6 levels, along with an increase in lean body mass. Another 12-week circuit training study by Jung et al. (39) in 2024 further confirmed these findings and, for the first time, observed a significant enhancement in blood flow-mediated vasodilation, suggesting improved vascular endothelial function. Balachandran et al. employed a high-speed circuit training protocol at 50%−80% of 1-RM, emphasizing rapid concentric contractions. Following the intervention, lower-body power output increased significantly by 41%, perceived exertion decreased, and physical function scores improved. All three studies confirmed that circuit training has a positive effect on improving body composition and cardiovascular metabolic risk. Compared to conventional circuit training, high-speed circuit training maximizes power output by optimizing the load, offering greater advantages in enhancing lower-limb explosive strength and the exercise experience, but it demands higher levels of movement control; conventional circuit training, on the other hand, has more robust evidence regarding improvements in body composition, metabolic and cardiovascular risk markers, muscle mass, and biomarkers, which may be related to its stable moderate-intensity load and longer duration per exercise session. Similarly, Meng et al. (44) conducted a 12-week program of combined aerobic and resistance exercise in 60 elderly patients on maintenance hemodialysis (SO). The sessions were held three times weekly, 30 min to 2 h after the start of dialysis, and consisted of a 5-min warm-up, 18 min of resistance training, 5–10 min of rest intervals, and 15–20 min of aerobic exercise on an air bike. Improvements in outcome measures demonstrated multisystem effects. Regarding body composition, the intervention group showed a significant decrease in body fat percentage, and VFA was significantly reduced at both 6 and 12 weeks. In terms of muscle strength, grip strength significantly improved after 12 weeks. Regarding muscle mass and biomarkers, SMI showed a trend toward improvement. In terms of quality of life, the KDQ score significantly improved after 12 weeks. A cross-comparison reveals that circuit training, with its alternating resistance and aerobic exercise pattern and shorter rest periods, provides more robust evidence for improving body fat percentage and vascular endothelial function, making it suitable for older adults in community settings and long-term care facilities (39, 44); in contrast, combined aerobic and resistance exercise, delivered as a synchronized intervention during dialysis, offers unique advantages in reducing body fat percentage and improving grip strength, making it particularly suitable for specific populations such as those undergoing maintenance hemodialysis (44). For elderly patients with SO, exercise interventions should be individually tailored based on population characteristics. Community-dwelling individuals may prioritize circuit training, while dialysis patients may adopt aerobic and resistance exercise combined during dialysis. Future research is recommended to explore optimal combinations of exercise intensity and timing during dialysis, in conjunction with nutritional interventions such as protein supplementation, to maximize training adaptability (63).

Adequate physical activity, by maintaining muscle mass and function, regulating fat metabolism, and improving systemic inflammation, serves as the core component of a foundational exercise intervention strategy for elderly patients with SO. Furthermore, it creates favorable metabolic conditions that may enhance the effectiveness of subsequent combined nutritional and exercise interventions. Ribeiro Santos et al. (42) conducted a 24-month prospective cohort study of 395 community-dwelling older adults, assessing physical activity levels *via* self-reported questionnaires, body composition using DXA, muscle strength using a dynamometer, and physical function using a walking test. The results showed that among older adults with insufficient activity in the occupational domain, those with reduced gait speed had a significantly increased risk of sarcopenia, and those with reduced muscle strength also faced a higher risk of sarcopenia; among older adults with insufficient activity in the transportation domain, those with reduced muscle mass, reduced gait speed, and high body fat all had a significantly increased risk of sarcopenia. These findings confirm that physical activity in different domains plays a differentiated role in improving outcome measures for patients with sarcopenia. Daily activities in the occupational domain—primarily involving sustained, low-intensity activities such as housework—have a protective effect on maintaining muscle strength and gait speed and preventing sarcopenia; walking or cycling in the transportation domain plays a key role in reducing the risk of SO by increasing overall energy expenditure, improving fat distribution, and promoting lower-limb function. It is worth noting that a study by Santos et al. (42) suggests that the protective effects of physical activity are domain-specific: occupational activities are more critical for preventing sarcopenia, while transportation activities are more effective for preventing SO. This suggests that older adults with SO should be encouraged to increase sustained activities such as daily housework; for populations at high risk of SO, emphasis should be placed on participation in transportation-related activities such as walking and cycling.

Compared to the general protective effects of daily physical activity, a moderate-intensity physical activity program, through a multi-component training model, provides a more targeted intervention strategy for older adults with sarcopenia and obesity. Gutiérrez-López et al. (43) implemented a 3-month moderate-intensity exercise program for older women with sarcopenic obesity, consisting of three 60-min sessions per week, each including a 10-min warm-up and flexibility exercises, 20 min of moderate-intensity aerobic exercise, and 20 min of low-intensity isometric exercise. Improvements in outcome measures demonstrated multidimensional effects: regarding body composition, the sarcopenic-obese group showed a significant decrease in body fat while maintaining muscle mass; regarding muscle strength, both groups exhibited significant improvements; regarding physical function, both the Timed Up and Go (TUG) test and gait speed improved significantly, with the sarcopenic-obese group's gait speed after the intervention becoming comparable to that of the healthy group. A cross-comparison revealed (42), both daily physical activity and moderate-intensity structured exercise can improve health outcomes in older adults with sarcopenic obesity, but the intervention mechanisms and dimensions of benefit differ. Due to its low barrier to entry and high adherence, daily physical activity provides long-term protective effects in maintaining SMI, grip strength, and gait speed, while occupational and transportation-related activities have domain-specific effects on preventing sarcopenia and the risk of frailty (42); moderate-intensity structured exercise, through a multi-component training model, has stronger evidence for reducing oxidative damage, enhancing antioxidant defenses, and improving physical function (43). Therefore, for older adults with sarcopenic obesity, daily physical activity should serve as a foundational intervention, while moderate-intensity structured exercise should act as an enhancement strategy, forming a combined intervention model of daily activity and structured exercise.

Posture, Strength, and Movement (PSM) training provides a systematic functional rehabilitation program for elderly patients with sarcopenic obesity by integrating multiple elements such as muscle strengthening, balance control, and motor coordination. Ferhi et al. (45) conducted a 24-week PSM training program for 40 elderly patients with sarcopenic obesity, consisting of two 60-min sessions per week. The training content was dynamically adjusted based on training load and included warm-ups, motor skill training, strength and posture training, and cool-down activities. The results showed that the intervention group experienced a significant increase in LBM, a significant decrease in FBM, and significant improvements in grip strength and gait speed. These findings suggest that PSM training may be associated with improvements in body composition, muscle strength, and physical function. The advantage of this training model lies in its organic integration of strength training with postural control and motor skill training; it stimulates muscle synthesis through resistance training while optimizing neuromuscular control efficiency through balance and coordination training. Notably, the post-intervention increase in gait speed was accompanied by an increase in grip strength, suggesting that PSM training may achieve functional optimization by enhancing neuromuscular coordination rather than simply increasing muscle mass (64). Future studies could further explore the dose-response relationship of different training components and incorporate additional functional measures to comprehensively evaluate the intervention's effects.

Power-strength training, by emphasizing high-speed concentric contractions to optimize neuromuscular recruitment efficiency, provides a targeted functional improvement program for elderly patients with sarcopenic obesity. Polo-Ferrero et al. (65) conducted a 32-week power-strength training program for 40 community-dwelling elderly women with SO. The training was performed three times weekly for 50 min per session, with training load dynamically adjusted based on perceived exertion, focusing on the rapid concentric contraction phase of lower-limb multi-joint movements. The results showed that the intervention group experienced significant increases in SMI and ASM, significant decreases in body fat percentage, VFA, and WC, and a significant improvement in gait speed. These findings confirm that power-strength training has a positive effect on improving body composition, muscle strength, and physical function. Notably, 35.7% of participants no longer met the diagnostic criteria for SO after the intervention, suggesting that power-strength training not only delays functional decline but also has the potential to reverse the disease state (65). Future studies may further explore the applicability of power-based strength training in men and in individuals with SO at different fitness levels, and combine it with nutritional interventions to optimize improvements in body composition.

### 4.2 Implementation methods and effects of nutritional interventions

Although the number of studies on nutritional intervention alone in this review is limited, with a primary focus on dietary behavior adjustments (49, 50), by analyzing the nutritional components in exercise-nutrition combined intervention studies, we can identify key nutritional strategies for SO in older adults. Existing evidence suggests that simple calorie restriction carries risks in SO management, whereas a “protein-priority” nutritional strategy combined with specific dietary behavior adjustments may be a superior approach.

#### 4.2.1 Protein thresholds and leucine-triggered mechanisms

Older adults often face “anabolic resistance,” meaning they require higher protein intakes than younger individuals to trigger muscle protein synthesis. In the combined intervention studies included in this review, protein supplementation doses typically ranged from 1.2 to 1.8 g/kg/day (52, 54, 55, 57). A study by Nilsson et al. (61) specifically noted that whey protein and casein, which are included in compound nutritional supplements, are rich in leucine and can effectively activate the mTOR signaling pathway, the molecular basis for reversing muscle loss. Based on current evidence, nutritional interventions for patients with SO should not focus solely on total caloric intake but should place greater emphasis on protein quality and dosage to maximize the efficiency of muscle protein synthesis.

#### 4.2.2 Synergistic effects of micronutrients

In addition to macronutrients, the role of micronutrients in the management of SO cannot be overlooked. In a study by Nilsson et al., a compound formulation containing vitamin D3 and fish oil was used. Vitamin D not only regulates calcium and phosphorus metabolism but also acts directly on type II muscle fibers to improve muscle contraction function (66); meanwhile, the omega-3 fatty acids in fish oil may help improve anabolic sensitivity in older adults through their anti-inflammatory properties (67).

#### 4.2.3 Age-specific risks associated with dietary behavior

Notably, a long-term cohort study by Lee et al. (50) revealed an interesting phenomenon: among individuals aged 65 and older, excessively slow eating speed was associated with an increased risk of sarcopenia (HR 1.603, 95% CI 1.119–2.298) and did not reduce the risk of obesity. The study noted that slow eating is associated with low energy intake and elevated postprandial GLP-1 and cholecystokinin concentrations. This may reduce total intake per meal by prolonging the sensation of fullness, thereby increasing the risk of protein-energy malnutrition. This finding complements the perspective proposed by Yin et al. (49) regarding the optimization of dietary quality through behavioral interventions. The latter study employed a moderate 12% calorie restriction combined with a protein intake of 1.2–1.5 g/kg/day; however, SMI still decreased following the intervention. This suggests that when guiding patients with SO on “healthy eating,” we must strike a delicate balance between “calorie control” and “ensuring adequate intake” to avoid exacerbating sarcopenia due to excessive dietary restriction.

### 4.3 Implementation and effects of combined exercise-nutrition interventions

Whole-body electrical muscle stimulation combined with protein supplementation provides a low-joint-load, time-efficient intervention for elderly patients with sarcopenic obesity through the synergistic effects of involuntary muscle contractions and optimized nutritional supply (68). Since 2016, Kemmler et al. (52, 54–57) have conducted a series of studies on this combined intervention, with their intervention protocols and outcome measures showing a trend toward gradual optimization. In a 2016 study of elderly women with SO (52), Kemmler et al. (52, 54–57) conducted a 26-week intervention involving weekly WB-EMS sessions and protein supplementation to achieve a total daily intake of 1.2–1.4 g/kg of body weight. The results showed significant improvements in sarcopenia Z-scores, skeletal muscle mass index, gait speed, grip strength, and body fat percentage, along with concurrent improvements in metabolic syndrome Z-scores, high-density lipoprotein cholesterol, and mean arterial pressure. In a 2018 study of elderly male patients with SO (55), Kemmler et al. (52) adjusted the intervention period to 16 weeks, increased the weekly training frequency to 1.5 sessions, and raised protein intake to 1.7–1.8 g/kg body weight/day. The results showed significant improvements in body fat percentage, trunk fat mass, waist circumference, triglycerides, and skeletal muscle mass index, with effect sizes generally higher than those reported in the earlier study of women. This series of studies suggests that the combination of WB-EMS and protein supplementation has led to positive changes in body composition, muscle strength, physical function, and metabolic and cardiovascular risk markers in patients with SO (52–57). Compared to studies in women, higher doses of protein supplementation and more frequent WB-EMS training in men may have further enhanced the effects of fat loss and metabolic improvement, whereas the longer intervention periods in women's studies resulted in more sustained improvements in functional measures such as gait speed and grip strength (52–57). The potential mechanisms underlying this combined intervention may include electrical stimulation directly activating deep muscle fibers, compensating for the limited voluntary contraction capacity in older adults (69), while optimized protein intake provides sufficient energy for muscle synthesis; together, these factors synergistically promote muscle mass gain and fat loss. Future studies should further explore the impact of gender differences on the response to the intervention and optimize individualized protocols for current intensity and protein dosage.

Compared to dietary interventions alone, a combination of compound nutritional supplements and exercise can simultaneously optimize muscle, fat, and metabolic markers. Nilsson et al. (61) conducted a 12-week study involving 20 older men who were overweight or obese and had at least one risk factor for metabolic syndrome. The study combined daily home-based walking with a compound nutritional supplement (containing whey protein, casein, creatine, vitamin D3, calcium, and fish oil).The results showed that the intervention group experienced significant improvements in limb lean body mass, body fat percentage, limb lean body mass index, and grip strength. This compound formulation promotes muscle protein synthesis by providing highly bioavailable protein; in combination with creatine, vitamin D3, and fish oil, it synergistically improves metabolic inflammation and bone health (70), offering a multi-target intervention strategy for patients with SO.

Exercise combined with nutritional intervention, through the synergistic effects of active training and multi-component nutritional supplementation, provides a comprehensive functional improvement program for elderly patients with sarcopenic obesity. Kim et al. (58) conducted a 3-month intervention involving 139 community-dwelling elderly Japanese women with SO, using a 2 × 2 factorial design. The exercise group underwent comprehensive training twice weekly for 60 min per session, including chair exercises, resistance band training, hydraulic machine training, and 12 min of aerobic exercise on a stationary bicycle; the nutrition group consumed a tea beverage fortified with 3.0 grams of leucine-enriched essential amino acids and 540 mg of catechins daily. Results showed that the exercise-plus-nutrition group exhibited significant improvements in body fat mass, trunk fat mass, limb fat mass, knee extension strength, gait speed, stride length, average daily step count, leptin, and vitamin D levels (58). Similar to Kemmler's study, the advantage of this combined intervention lies in the fact that active exercise simultaneously activates neuromuscular coordination and cardiopulmonary function, while amino acids provide the foundation for muscle synthesis and catechins enhance fat loss by promoting fat oxidation (71). The synergy of these three components achieves multidimensional optimization of body composition and function. However, compared with WB-EMS, the active exercise protocol in this study places greater emphasis on the comprehensive development of neuromuscular coordination and cardiorespiratory endurance, resulting in more comprehensive improvements in walking ability indicators such as gait speed and stride length. The two approaches are complementary in terms of intervention methods, providing differentiated treatment options for SO patients with varying levels of physical fitness.

Resistance training combined with whey protein supplementation provides dual benefits of increased muscle mass and improved metabolism for elderly patients with sarcopenic obesity by optimizing the environment for muscle protein synthesis. Nabuco et al. (59) conducted a 12-week program of resistance training combined with daily supplementation of 35 g of whey protein in 26 elderly female patients with sarcopenic obesity. The results showed a significant increase in lean soft tissue in the limbs and a significant decrease in total body fat, trunk fat, and interleukin-6. Han et al. (60) conducted a 12-week program of resistance training combined with daily supplementation of 20 g of whey protein in 75 elderly women with SO and type 2 diabetes. They observed significant improvements in glycated hemoglobin, insulin resistance index, skeletal muscle mass in the limbs, grip strength, and time to complete five sit-to-stand tests. The advantage of this combined intervention lies in the fact that whey protein is rich in leucine, which effectively activates muscle protein synthesis signaling pathways (72), while resistance training further enhances anabolic effects through mechanical stimulation. Compared with resistance training alone, the combination with whey protein supplementation provided additional benefits in improving skeletal muscle mass in the limbs; however, no synergistic effect was observed regarding improvements in blood glucose and insulin resistance, suggesting that resistance training alone may be sufficient to drive metabolic improvements in individuals with diabetes.

### 4.4 The energy balance paradox and challenges in nutritional intervention

The pathophysiological core of sarcopenic obesity lies in the coexistence of “low muscle” and “high fat,” which presents a unique energy balance paradox for nutritional interventions. Traditional obesity management emphasizes a caloric deficit to reduce body weight, but this strategy is often accompanied by a loss of muscle mass, which may further exacerbate sarcopenia; conversely, traditional sarcopenia management emphasizes adequate energy intake to support muscle protein synthesis, but this may lead to fat accumulation and worsen obesity.

The studies included in this review attempted to address this contradiction through moderate calorie restriction combined with high protein intake. Yin et al. (49) explored an intervention protocol involving 12% calorie restriction combined with 1.2–1.5 g/kg/d of protein; however, even under these conditions, a downward trend in skeletal muscle mass index (SMI) was observed, which precisely underscores the challenge posed by this paradox. In contrast, the research by Kim et al. (58), which explored the addition of functional ingredients (such as catechins) to a moderate calorie-restricted diet, offers new insights into achieving fat loss while preserving muscle mass by modulating metabolic pathways.

These findings suggest that future nutritional intervention strategies should shift from simple “calorie control” to “nutrient allocation”—that is, significantly increasing the proportion of energy derived from protein and optimizing macronutrient composition while maintaining a mild caloric deficit or energy balance. Future research should further explore the specific effects of different nutritional redistribution models (e.g., high-protein/low-carbohydrate diet vs. high-protein/Mediterranean diet) on body composition in patients with SO, in order to develop more precise, individualized nutritional prescriptions.

## 5 Conclusions

This scoping review systematically mapped the evidence landscape of nutrition, exercise, and combined interventions on health outcomes in older adults with SO, providing systematic evidence support for the core value of nutritional and exercise strategies in managing this population. Existing evidence indicates that optimizing protein intake and nutritional composition, regular exercise, and their synergistic combined interventions can all improve patients' body composition, muscle function, and metabolic health. Among these, protein supplementation centered nutritional interventions and combined exercise nutrition interventions reported the broadest positive effects on body composition and metabolic indicators; however, the evidence primarily originated from a single research team, and independent validation remains insufficient. Although exercise training reported the most consistent benefits in muscle function improvement, this review proposes that nutritional interventions, for example, adequate high quality protein and specific amino acid supplementation, constitute the metabolic foundation of SO management, providing the necessary material conditions for the muscle synthesis effects of exercise interventions. Overall, the existing evidence for improving health outcomes in older adults with SO supports a strategy with nutritional intervention as the foundation and exercise intervention as the driver, while also revealing the fragmented status of this field characterized by diverse intervention protocols and scattered outcome measures.

Based on these findings, future research should focus on the following directions. First, researchers should establish a standardized core outcome measure set covering key domains such as body composition, muscle function, metabolic risk, and quality of life, to improve comparability across studies and evidence synthesis efficiency. Second, investigators should conduct dose response studies of nutritional interventions; after clarifying the optimal protein supplementation dosage and timing, they should further explore the synergistic roles of micronutrients in improving sarcopenic obesity. Third, researchers should optimize individualized intervention protocols by integrating patients' sarcopenia severity, obesity phenotypes, and cultural dietary backgrounds, to develop precise and context-specific nutrition and exercise prescriptions. Fourth, future studies should be conducted across more diverse geographic and cultural settings to validate the broad applicability of existing findings. Fifth, this review included only Chinese and English literature, potentially omitting relevant studies published in other languages; future research could consider incorporating literature in additional languages to expand evidence coverage. Furthermore, as a scoping review, this study aimed to map the existing evidence landscape rather than evaluate intervention effectiveness; therefore, we did not conduct formal risk of bias assessments on the included studies. These findings should be viewed as a synthesis of the current evidence landscape rather than a definitive conclusion on intervention effectiveness.

## Data Availability

The original contributions presented in the study are included in the article/supplementary material, further inquiries can be directed to the corresponding authors.
