# Supplementary material for: Optimizing health outcomes for older adults with sarcopenic obesity: a scoping review of nutrition, exercise and combined interventions
Source: Front Nutr. 2026 Oct 7;13:1936660. doi: 10.3389/fnut.2026.1936660 (PMC13684071; doi:10.3389/fnut.2026.1936660)
Supplement: Supplementary file 1 [file Supplementary_file_1.docx]

Appendix A

Appendix A.1 Search strategy

PubMed

((("sarcopenia"[MeSH Terms] OR "sarcopenia"[Title/Abstract] OR "sarcopenic"[Title/Abstract])

AND ("obesity"[MeSH Terms] OR "obesity"[Title/Abstract] OR "obese"[Title/Abstract] OR "overweight"[Title/Abstract] OR "body composition"[Title/Abstract])) OR ("sarcopenic obesity"[Title/Abstract] OR "sarcopenic obese"[Title/Abstract]))

AND ("aged"[MeSH Terms] OR "aged"[Title/Abstract] OR "older adult*"[Title/Abstract] OR "elderly"[Title/Abstract] OR "aging"[Title/Abstract] OR "ageing"[Title/Abstract])

AND (english[Language] OR chinese[Language])

Web of Science Core Collection

TS=((sarcopenia OR sarcopenic) AND (obesity OR obese OR overweight OR "body composition")) OR TS=("sarcopenic obesity" OR "sarcopenic obese")

AND TS=("aged" OR "older adult*" OR elderly OR aging OR ageing)

AND LA=(English OR Chinese)

CINAHL

((MH "Sarcopenia") OR TI (sarcopenia OR sarcopenic) OR AB (sarcopenia OR sarcopenic))

AND ((MH "Obesity") OR TI (obesity OR obese OR overweight OR "body composition") OR AB (obesity OR obese OR overweight OR "body composition")) OR (TI ("sarcopenic obesity" OR "sarcopenic obese") OR AB ("sarcopenic obesity" OR "sarcopenic obese"))

AND ((MH "Aged") OR TI ("aged" OR "older adult*" OR elderly OR aging OR ageing) OR AB ("aged" OR "older adult*" OR elderly OR aging OR ageing))

AND (LA English OR LA Chinese)

Embase

('sarcopenia'/exp OR 'sarcopenia':ti,ab OR 'sarcopenic':ti,ab)

AND ('obesity'/exp OR 'obesity':ti,ab OR 'obese':ti,ab OR 'overweight':ti,ab OR 'body composition':ti,ab) OR ('sarcopenic obesity':ti,ab OR 'sarcopenic obese':ti,ab)

AND ('aged'/exp OR 'aged':ti,ab OR 'older adult*':ti,ab OR 'elderly':ti,ab OR 'aging':ti,ab OR 'ageing':ti,ab)

AND ([english]/lim OR [chinese]/lim)

CNKI

(SU='肌少症' OR SU='肌肉减少症' OR SU='骨骼肌减少症' OR SU='少肌症' OR TI='肌少症' OR TI='肌肉减少症' OR TI='骨骼肌减少症' OR TI='少肌症')

AND (SU='肥胖' OR SU='超重' OR SU='体成分' OR SU='脂肪' OR TI='肥胖' OR TI='超重' OR TI='体成分' OR TI='脂肪')

AND (SU='老年人' OR SU='老年' OR SU='社区老年人' OR SU='高龄' OR TI='老年人' OR TI='老年' OR TI='社区老年人' OR TI='高龄')

Wanfang

主题:(肌少症 OR 肌肉减少症 OR 骨骼肌减少症 OR 少肌症)

AND 主题:(肥胖 OR 超重 OR 体成分 OR 脂肪)

AND 主题:(老年人 OR 老年 OR 社区老年人 OR 高龄)

VIP

M=(肌少症 OR 肌肉减少症 OR 骨骼肌减少症 OR 少肌症)

AND M=(肥胖 OR 超重 OR 体成分 OR 脂肪)

AND M=(老年人 OR 老年 OR 社区老年人 OR 高龄)

SinoMed

("肌少症"[智能题名] OR "肌肉减少症"[智能题名] OR "骨骼肌减少症"[智能题名] OR "少肌症"[智能题名])

AND ("肥胖"[智能题名] OR "超重"[智能题名] OR "体成分"[智能题名] OR "脂肪"[智能题名])

AND ("老年人"[智能题名] OR "老年"[智能题名] OR "社区老年人"[智能题名] OR "高龄"[智能题名])
